# Crucial role of serum response factor in renal tubular epithelial cell epithelial-mesenchymal transition in hyperuricemic nephropathy

**DOI:** 10.18632/aging.102479

**Published:** 2019-11-27

**Authors:** Long Zhao, Chenyu Li, Bin Zhou, Congjuan Luo, Yanfei Wang, Lin Che, Jun Zhao, Yan Xu

**Affiliations:** 1 Department of Nephrology, The Affiliated Hospital of Qingdao University, Qingdao 266003, China; 2 Department of Nephrology, Shandong Weifang People’s Hospital, Weifang 261041, China

**Keywords:** serum response factor, slug, renal tubular epithelial cells, epithelial-mesenchymal transition, hyperuricemic nephropathy

## Abstract

Objective: To explore the regulation and function of serum response factor (SRF) in epithelial-mesenchymal transition (EMT) in renal tubular epithelial cells (TECs) in hyperuricemic nephropathy (HN).

Results: In NRK-52E cells treated with UA and renal medulla tissue samples from hyperuricemic rats, SRF, fibronectin, α-SMA and FSP-1 expression was upregulated, while ZO-1 and E-cadherin expression was downregulated. SRF upregulation in NRK-52E cells increased slug expression. Blockade of SRF by an SRF-specific siRNA or CCG-1423 reduced slug induction and protected TECs from undergoing EMT both *in vitro* and *in vivo*.

Conclusion: Increased SRF activity promotes EMT and dysfunction in TECs in HN. Targeting SRF with CCG-1423 may be an attractive therapeutic strategy in HN.

Methods: The expression of SRF, mesenchymal markers (fibronectin, α-SMA, and FSP-1), epithelial markers (ZO-1 and E-cadherin) and was examined in rat renal TECs (NRK-52E cells) or renal medulla tissue samples following uric acid (UA) treatment. SRF overexpressed with pcDNA-SRF plasmid and suppressed by CCG-1423 (a small molecule inhibitor of SRF) to study how SRF influences EMT in TECs in HN. Oxonic acid (OA) was used to establish HN in rats.

## INTRODUCTION

In recent years, the prevalence of hyperuricemia has increased rapidly worldwide, especially in coastal areas. With the changes in dietary structure, purine intake continues to increase [1]. The prevalence of hyperuricemia in American adults achieved 21.4% (21.6% in female and 21.1% in male) according to the National Health and Nutrition Examination Survey (NHANES) 2007–2008 [2]. From 2000 to 2014, as shown by a recent authoritative meta-analysis, the pooled prevalence of hyperuricemia of Chinese mainland was 13.3% (7.9% in female and 19.4% in male) [3]. Hyperuricemia is a serious health hazard that can lead to gouty arthritis, coronary heart disease, hypertension, diabetes and other metabolic syndrome-related diseases [4]. The kidneys are the most important organ for the excretion of uric acid (UA), and they are also vulnerable to UA damage [5, 6]. Patients with hyperuricemia have a 2.14 times higher risk of chronic kidney disease than those with normal UA levels [7]. Therefore, it is of great clinical significance to explore the mechanism underlying hyperuricemic nephropathy (HN).

Renal tubular epithelial cells (TECs) are the renal cells most vulnerable to UA injury [8, 9]. Current studies show that hyperuricemia can cause renal tubule damage via a variety of mechanisms, such as causing oxidative stress [10], inhibiting nitric oxide synthesis [11], inhibiting epithelial cell proliferation [12], activating inflammation [13], inducing vascular smooth muscle cell (SMC) proliferation [14], and inducing epithelial-mesenchymal transition (EMT) in TECs [15, 16], which consequently cause renal structure and function abnormalities and eventually renal fibrosis. However, the mechanism of renal injury induced by hyperuricemia has not been fully elucidated, and there is no targeted therapy for HN. Therefore, in-depth discussion on how hyperuricemia resulted in renal damage can provide a new idea for effective prevention and treatment of HN.

EMT of TECs is identified as the initiator of tubulointerstitial fibrosis process. When TECs is undergoing EMT, the expression of epithelial markers is declined, while mesenchymal marker levels are upregulated. The HLH family, Zeb and Snail are critical transcriptional suppressors of many genes including E-cadherin which are relevant to EMT [16]. Nevertheless, those above cytokines could not totally illuminate the specific pathogenesis underlying TEC EMT. Therefore, novel transcription factors related to EMT of TECs is still required to be identified.

During peritoneal dialysis, SRF/Snail signaling pathway activation may play a role in the progression of peritoneal membrane fibrosis [17]. It was also reported that SRF had significant roles in progressive carcinoma, specifically in the process of EMT and in the ability of migration and invasion in many cancers, for example, prostate cancer [18], gastric carcinoma [19], hepatocellular carcinoma [20]. However, how SRF regulates renal tubular injury in HN remains mostly unknown.

Herein, in this study, it is investigated whether SRF played a role in HN rat model and immortalized rat TECs. In addition, it is also evaluated whether there is a therapeutic potential in SRF/slug pathway.

## RESULTS

### UA increased SRF expression and mediated EMT of NRK-52E cells

**Figure 1:**
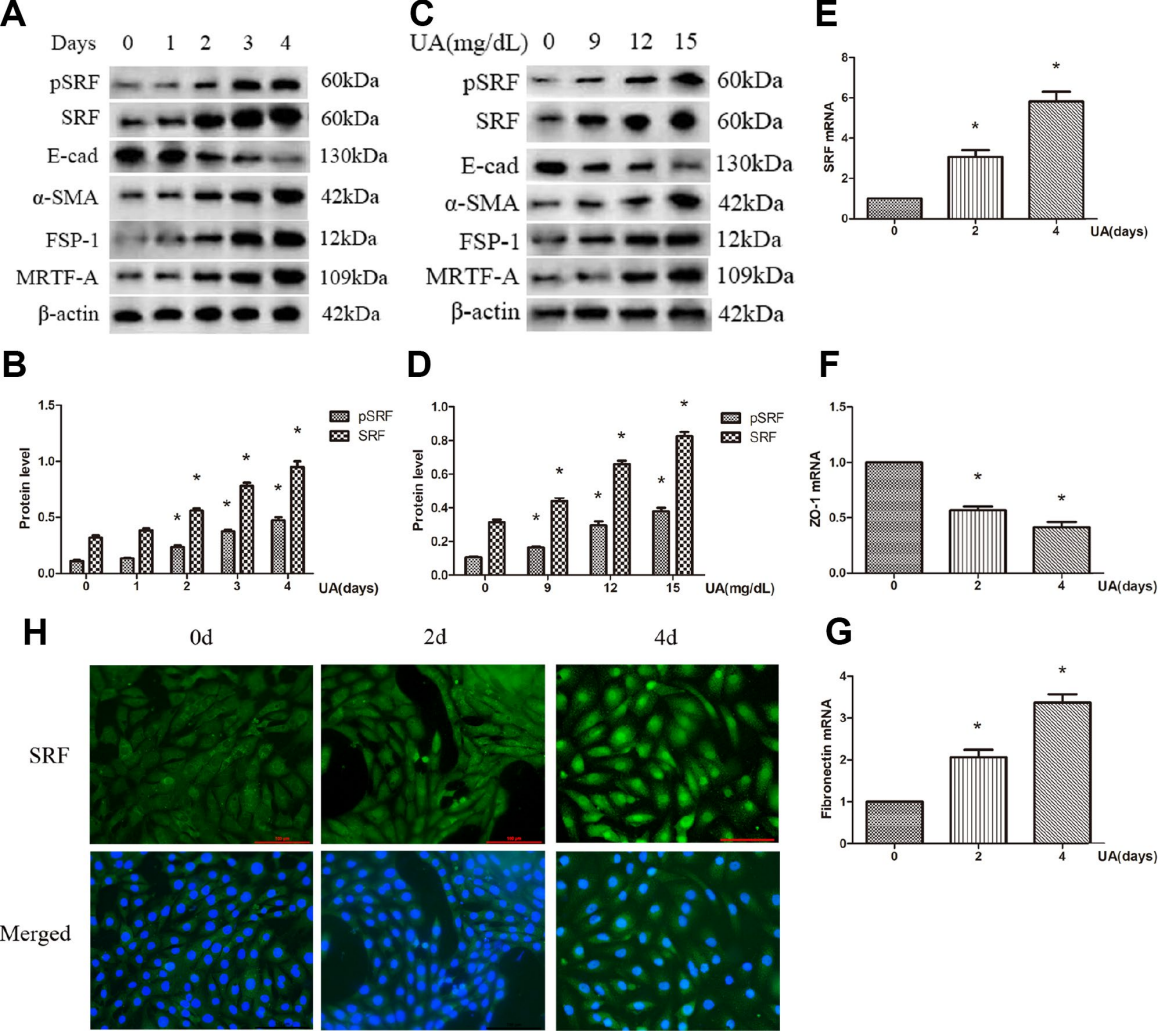
**Uric acid (UA) induced epithelial-mesenchymal transition (EMT) and serum response factor (SRF) expression upregulation in NRK-52E cells.** (**A**) Western blot analysis showing that UA (15 mg/dL) mediated the upregulation of phosphorylated SRF (pSRF), SRF, α-smooth muscle actin (α-SMA), and Fibroblast specific protein-1 (FSP-1) expression and the downregulation of E-cadherin expression in a time-dependent manner. (**C**) Western blot analysis showing that UA mediated the upregulation of pSRF, SRF, α-SMA, and FSP-1 expression and the downregulation of E-cadherin expression in a dose-dependent manner at 48 h. (**B**, **D**) Quantitative determination of relative SRF levels. (**E**–**G**) Quantitative RT-PCR analysis of SRF, ZO-1 and fibronectin (FN) mRNA expression. (**H**) Immunofluorescence staining for SRF at an original magnification of 400×. *P<0.05 versus the control group. All experiments were repeated three times.

Both the protein and mRNA expression of SRF were increased after UA treatment by a dose-dependent and time-dependent manner. What is more, the expression of phosphorylated SRF (pSRF) was also upregulated, which is taken for the biochemically activated form of SRF (Figure 1A–1E). In addition, the treated NRK-52E cells tended to undergo EMT. As described in Figure 1A–1D, UA suppressed ZO-1 and E-cadherin expression and induced FSP-1, α-SMA and FN upregulation by a time-dependent and dose-dependent manner. As measured by immunofluorescence staining, after UA treatment, the expression of SRF was upregulated, and SRF obviously translocated from the cytoplasm to the nucleus of NRK-52E cells as well (Figure 1H). Interestingly, the expression of MRTF-A was also upregulated in NRK-52E cells after UA stimulation, which is an upstream activator of SRF (Figure 1A and 1C).

### SRF overexpression induced EMT and migration of NRK-52E cells

**Figure 2:**
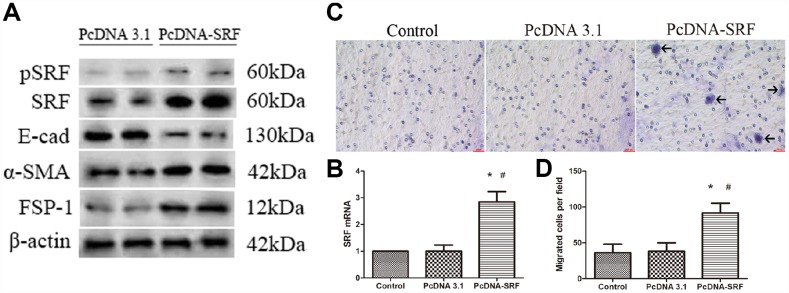
**SRF overexpression induced EMT and migration in NRK-52E cells.** (**A**) Western blot analysis showing the induction of SRF, α-SMA, and FSP-1 expression and a reduction in E-cadherin expression. (**B**) Quantitative RT-PCR analysis of SRF mRNA expression. (**C**) Representative micrographs of the transwell chamber migration assay at an original magnification of 200×. Arrowheads indicate some of the migrated cells. Bar=50 μm. (**D**) Quantitative analysis of the number of migrated NRK-52E cells per field in three groups. *P<0.05 versus the control group; ^#^*P*<0.05 versus the pcDNA3.1 group. All experiments were repeated three times.

As described in Figure 2A and 2B, both the mRNA and protein expression of SRF was upregulated in NRK-52E cells transfected with empty pcDNA plasmids or pcDNA-SRF plasmids. As demonstrated by western blot in Figure 2A, ectopic expression of SRF elevated FSP-1 and α-SMA levels and decreased E-cadherin level in NRK-52E cells. Those above results indicate that SRF overexpression in NRK-52E cells results in a phenotypic change reminiscent of EMT. In addition, SRF overexpression obviously increased the shift of NRK-52E cells cross transwell filter membranes (Figure 2C and 2D), pointing that EMT induced by SRF alone was sufficient to increase the migratory capacity of the NRK-52E cells.

### Suppression of SRF preserved the phenotype of NRK-52E cells after UA treatment

**Figure 3:**
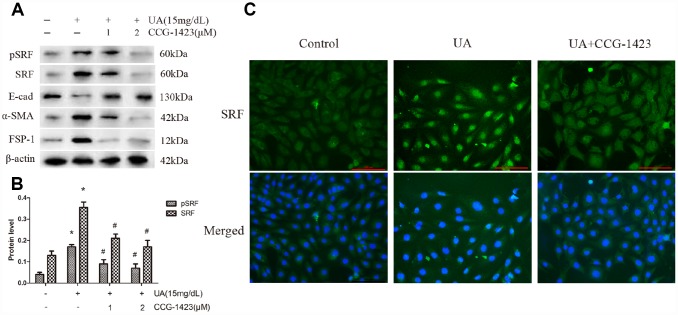
**Inhibition of SRF preserved the phenotype of NRK-52E cells after UA stimulation *in vitro.*** NRK-52E cells were pretreated with CCG-1423 (1 μM or 2 μM) or DMSO for 1 h, followed by treatment with UA (15 mg/dL) for 72 h. (**A**) Protein expression of pSRF, SRF, E-cadherin, α-SMA and FSP-1 measured by western blot analysis. (**B**) Quantitative determination of the pSRF and SRF protein levels. (**C**) Immunofluorescence staining for SRF at an original magnification of 400×. CCG-1423 was used at a concentration of 2 μM. *P<0.05 versus the control group; ^#^*P*<0.05 versus the UA group. All experiments were repeated three times.

For the purpose of evaluating if the suppression of SRF protects NRK-52E cells from UA injury, CCG-1423 was applied, which is a MRTF/SRF pathway suppressor. The mechanism, specificity and potency of CCG-1423-mediated inhibition of the MRTF/SRF signaling pathway were confirmed previously [21–23]. CCG-1423 downregulated SRF and pSRF levels by a dose-dependent manner after 72h of UA treatment in NRK-52E cells (Figure 3A and 3B). As described in Figure 3C, CCG-1423 suppressed the expression and the translocation of SRF in NRK-52E cells stimulated by UA as well. In addition, simultaneous treatment of CCG-1423 and SRF overexpression obviously blocked the upregulation in FSP-1 and α-SMA and the downregulation in E-cadherin as well, mostly preventing NRK-52E cells from undergoing EMT (Figure 3A).

### Inhibition of SRF suppressed the upregulation of slug expression *in vitro*

**Figure 4:**
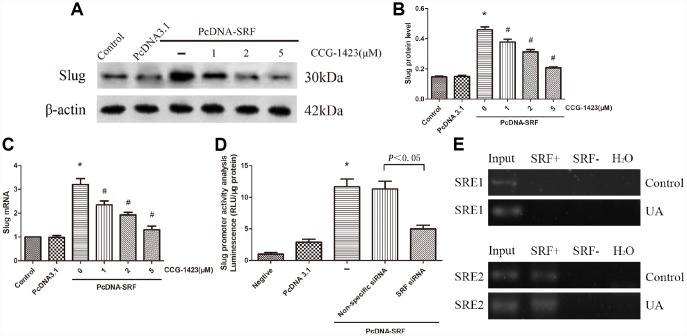
**Inhibition of SRF suppressed slug expression upregulation in vitro**. (**A**–**C**) NRK-52E cells were transfected with pcDNA3.1-SRF or empty pcDNA3.1 vectors. After 24 h, the cells were incubated with different doses of CCG-1423 for another 24 h. The protein and mRNA expression levels of slug were measured by western blot analysis and quantitative RT-PCR. (**D**) Slug promoter activity was analyzed by measuring luminescence. (**E**) Chromatin immunoprecipitation was used to examine SRF binding to the slug promoter in NRK-52E cells. Reaction controls included immunoprecipitations performed using a nonspecific IgG monoclonal antibody; PCR was performed using whole cell genomic DNA (Input). N=6 in each group. *P<0.05 versus the pcDNA3.1 group; #P<0.05 versus the pcDNA-SRF group. All experiments were repeated three times.

An experiment combining SRF overexpression and inhibition was carried out. Figure 4A–4C indicated that CCG-1423 dramatically suppressed the slug expression induced by SRF overexpression by a dose-dependent manner, suggesting that the suppression of SRF through CCG-1423 was able to abolish a critical transcription factor that regulates EMT under various conditions.

To further validate whether SRF directly regulates slug, an SRF-specific siRNA and luciferase assay were used. As described in Figure 4D, slug promoter activity was significantly increased by SRF overexpression and remarkably decreased by the SRF-specific siRNA, and these results were consistent with those produced by CCG-1423 treatment.

In order to explore how SRF regulates slug, ChIP assay was carried out to evaluate the binding of SRF to slug of NRK-52E cells. The predicted SRF binding sites in slug gene promoter regions were SRE1 and SRE2, which were both serum response elements (SRE). The threshold of positive SRF binding was fifty-fold enrichment. As shown in Figure 4E, SRF positively binded to SRE2 of NRK-52E cells and the binding signal was elevated in UA group, which is consistent with their increased expression of SRF.

### Suppression of SRF attenuate albuminuria and renal tubulointerstitial fibrosis in rats

**Figure 5:**
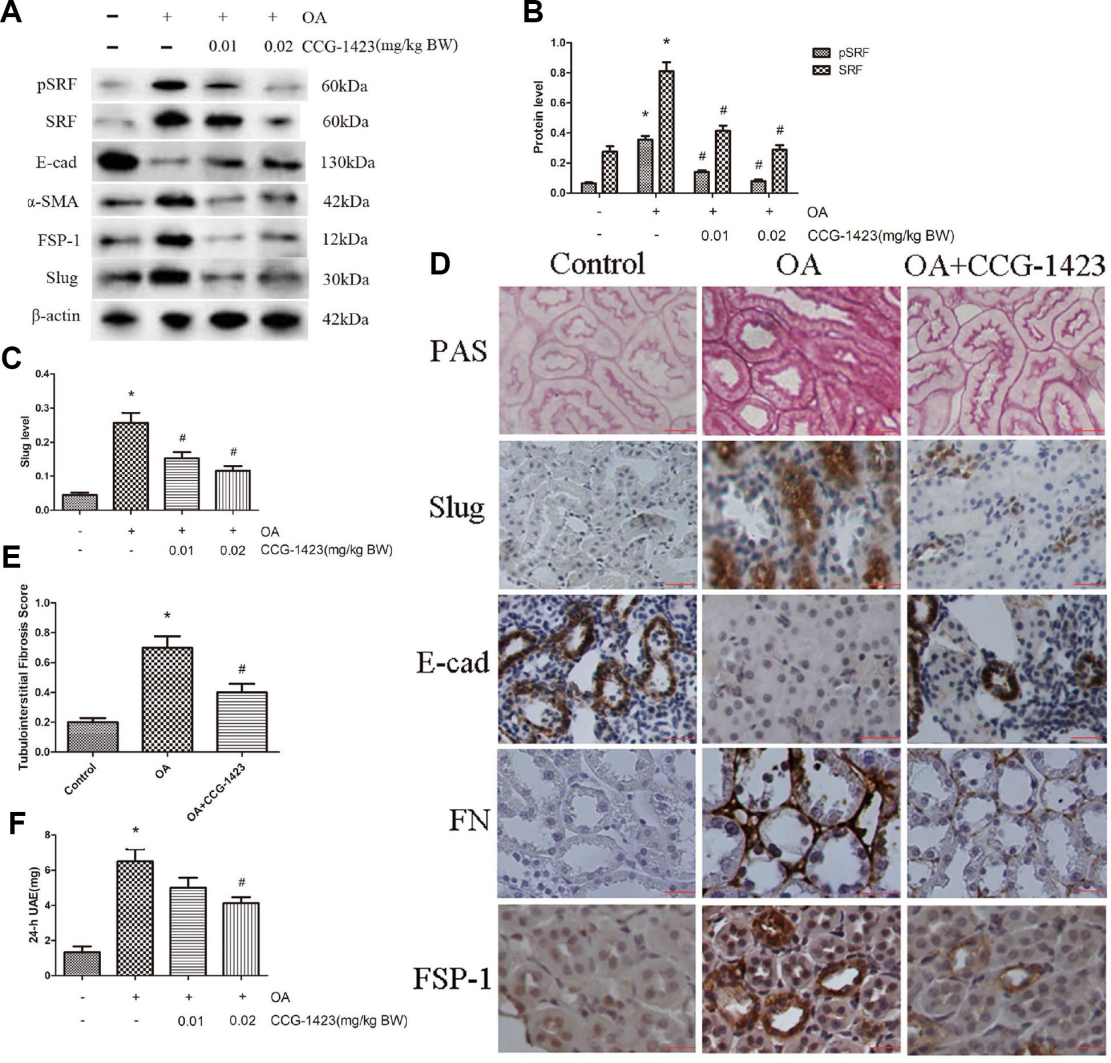
**Inhibition of SRF improved the epithelial phenotype of TECs, renal tubulointerstitial fibrosis and albuminuria *in vivo.*** (**A**–**C**) Protein expression levels of pSRF, SRF, E-cadherin, α-SMA, FSP-1 and slug measured by western blot and quantitative analyses. (**D**) Renal tubulointerstitial fibrosis in the three groups measured by PAS staining. Immunohistochemical staining for slug, E-cadherin, FN and FSP-1 in the three groups. (**E**) Tubulointerstitial fibrosis scores of the three groups. (**F**) Dose-dependent reduction in 24-h UAE in HN rats induced by CCG-1423 treatment. Bar=25 μm. N=6 in each group. *P<0.05 versus the control group; ^#^*P*<0.05 versus the OA group.

As shown in Table 1, the establishment of an HN model was verified by the results of biochemical analysis, which showed merely the serum albumin level was ameliorated in CCG-1423-treated HN rats as well. As shown in Figure 5A–5D, the suppression of SRF by CCG-1423 dramatically blocked the upregulation in FN, FSP-1, α-SMA, SRF, slug and the downregulation in E-cadherin of renal medulla tissue samples. PAS staining demonstrated there were renal tubulointerstitial fibrosis in the OA group, and tubulointerstitial fibrosis was significantly improved after CCG-1423 treatment for 6 weeks in the OA+CCG group compared with the OA group (Figure 5D and 5E). What is more, CCG-1423 improved albuminuria by a dose-dependent manner (Figure 5F). Compared with the vehicle control, 24-h UAE was significantly reduced by CCG-1423 at a dose of 0.02 mg/kg of BW in approximately 30%. Those findings suggested targeting SRF with a small molecular suppressor was able to ameliorate TEC EMT, renal tubulointerstitial fibrosis and albuminuria.

**Table 1:** Biochemical analysis of rats.

	**Control**	**OA**	**OA+CCG-1423**
Serum uric acid (mg/dL)	1.01±0.09	3.5±0.23^*^	3.4±0.28^*^
Scr (mg/dL)	0.41±0.03	0.92±0.07^*^	0.91±0.08^*^
BUN (mg/dL)	8.45±0.70	22±1.76^*^	21±1.66^*^
Serum albumin(g/L)	30.01±2.38	20.20±1.54^*^	28.70±1.66^#^

CCG-1423 was administrated at the concentration of 0.02 mg/kg BW. Scr: serum creatinine; BUN: blood urea nitrogen. Data were expressed as mean ± SEM. N=6 in each group. ^*^ *P* < 0.05 versus Control group, ^#^*P* < 0.05 versus OA group.

## DISCUSSION

A recent study established a vital role for SRF in the maintenance of podocyte structure and function, indicating a tight relation between kidney injury and SRF [24]. Remarkably, SRF were increase and activated by UA stimulation both *in vivo* (Figure 5) and *in vitro* (Figure 1). SRF expression was upregulated in renal medulla tissue samples from the HN rats (Figure 5), which was characterized by dramatic renal tubulointerstitial fibrosis. Of interest, the expression of MRTF-A was also increased in UA-induced NRK-52E cells (Figure 1). In consideration of MRTF-A was the major upstream regulator of SRF, the coinstantaneous induction of the expression of SRF and MRTF-A might result in amplified SRF activation in HN.

It has been shown that SRF is closely correlated to renal fibrosis in diabetic nephropathy and plays an important role in EMT in many types of renal cells, such as podocytes [24], TECs [25] and glomerular endothelial cells [26]. Moreover, TEC EMT is an important process in the progression of HN [16, 27]. Therefore, SRF overexpression in NRK-52E cells upregulated a lot of mesenchymal markers and inhibited a lot of epithelial markers as well. Those changes of cytoskeletal factors (α-SMA) and cell-cell adhesion factors (E-cadherin) can lead to albuminuria and impaired renal function. Interestingly, SRF overexpression increased NRK-52E cell motility and migration, which may be a result or a portion of EMT, resulting in functional nephron numbers decrease. To sum up, those findings indicated the increased SRF expression is competent to alter TEC phenotypes and mobility that are related to EMT and migration.

Although the specific pathogenesis by which SRF promotes EMT of TECs remains to be demonstrated, these mechanisms appear to be related to the ability to induce the expression of slug. Slug is known as a key regulating gene of EMT, which plays a significant part in fibrosis through reducing the expression of E-cadherin [16]. It has been shown SRF and slug are important mediators of TGF-β1-induced EMT [28]. Hence, forced expression of SRF dramatically increased slug level (Figure 4). Suppressing SRF through CCG-1423 inhibited UA-mediated TEC EMT, slug induction and 24-h UAE. Albumin, the dominant protein in the glomerular filtrate, is absorbed by TECs through receptor-mediated endocytosis, which is a necessary process for homeostasis. However, EMT could disrupt the endocytic function to promote albuminuria of HN [29]. In summary, these findings indicate crucial part for SRF/slug signaling pathway in promoting TEC EMT and disfunction.

CCG-1423 was reported to be a hopeful small molecular substances to prevent the progression of prostate cancer [30]. The present research showed SRF might also be an attractive treatment target point in HN. In HN rats, CCG-1423 ameliorated EMT, albuminuria and renal tubulointerstitial fibrosis by a dose-dependent manner, which is in line with the role of SRF *in vivo*. No drugs have been shown to treat HN by inhibiting EMT. However, this study demonstrates that CCG-1423 may be an attractive pharmacological compound for improving HN progression through blocking EMT, which may fill a gap in the HN field.

It should be underlined that the present study has several limitations and defects because we only used a hyperuricemia rat model established with a hepatic uricase inhibitor and an immortalized NRK-52E cell line. Whether these data can be extended to cultured primary TECs or a hyperuricemia mouse model established by knocking out the urate oxidase gene [1] remains to be illuminated. Moreover, whether our results can be expanded to HN of human remains to be addressed. Last but not least, MRTF-A has reported SRF-independent effects on cell proliferation and migration that could confound the results.

## CONCLUSIONS

In summary, this study has demonstrated that SRF expression is increased in UA-induced TECs, which may play a critical part in promoting dysfunction and EMT. Therefore, our findings demonstrate proof of the principle that the pharmacological compound targeting the SRF/slug signaling is a promising strategy of HN treatment.

## MATERIALS AND METHODS

Oxonic acid (OA; a hepatic uricase inhibitor) and UA were purchased from Sigma (St. Louis, MO, USA). Anti-β-actin (1:1000, BS1002) and anti-pSRF (1:1000, BS4177) antibodies were obtained from Bioworld Technology (Louis Park, MN, USA); anti-SRF antibody (1:500, sc-335) and anti-slug (1:500, sc-166902) antibodies were obtained from Santa Cruz (Santa Cruz, CA, USA); and anti-collagen I (1:1000, ab34710), anti-E-cadherin (1:1000, ab76055), anti-fibronectin (FN) (1:1000, ab2413), anti-α-smooth muscle actin (α-SMA) (1:1000, ab5694), anti-Fibroblast specific protein-1 (FSP-1) (1:1000, ab27957) and anti-MRTF-A (1:1000, ab49311) antibodies were obtained from Abcam (Cambridge, MA, USA). CCG-1423 was obtained from Cayman Chemical (Ann Arbor, MI, USA).

### Animal protocols

Male Sprague-Dawley rats (8 weeks of age and weighing 200–250 g) were purchased from the Animal Center of Qingdao University. Rats were given free access to water and food in the study. After 2 weeks of adaptation, the diet containing 2% OA was given to the rats. The animals were randomly stratified into three groups with equal average initial body weight (BW): (i) nonhyperuricemic normal rats (control group, n=6); (ii) OA-induced hyperuricemic rats (OA group, n=6); and (iii) OA-induced hyperuricemic rats treated with CCG-1423 diluted in a 1:100 solution of dimethyl sulfoxide (DMSO):phosphate-buffered saline (PBS) (OA+CCG group, n=12). CCG-1423 was administered by daily intraperitoneal injection at doses of 0.01 and 0.02 mg/kg of BW for 6 weeks beginning on the day after OA treatment. As a control, the same volume of the vehicle (100 μL/100 g of BW) was administered to the control and OA group animals. At 6 weeks, the rats were euthanized to assess renal function and histologically evaluate the kidneys. There was no mortality during the study period.

### Sample collection, biochemical analysis and immunohistochemical staining

These methods were described previously [26].

### Histopathological analysis

To assess renal interstitial fibrosis injury, kidney tissue samples were embedded in paraffin, and 3-μm-thick sections were prepared for periodic acid Schiff (PAS) staining to demonstrate fibrosis in the renal tissue samples. Tubulointerstitial fibrosis was analyzed after PAS staining and graded according to a semiquantitative scoring system ranging from 0 to 3 (0: normal, 1: mild, 2: moderate, and 3: severe) at 400× magnification in a total number of 100 tubules per section [31].

### Cell culture and treatment

NRK-52E cells were obtained from the American Type Culture Collection (ATCC, Manassas, VA, USA). Cells were cultivated at 37°C in DMEM (HyClone, Logan, UT, USA) supplemented with 10% fetal calf serum (Gibco, Langley, OK, USA) and 1% penicillin/ streptomycin (Gibco, Langley, OK, USA). NRK-52E cells were administrated with UA with or without CCG- 1423 (1, 2, or 5 μM) which was dissolved in DMSO. At the same time, the same volume of DMSO was given to the control group.

### Small-interfering RNA (siRNA) and a luciferase assay

An SRF-specific siRNA (5′-GACCTGCCTCAAC TCGCCAGAC-3′) was described previously [32]. Slug upstream regions, −450 to +164 relative to transcription start site, were cloned into pGL3-Basic vector (Promega, Madison, Wisconsin, USA), upstream of firefly luciferase coding sequence via PCR and subsequent ligation. Primers were as follows: Slug F, 5′-TTGTGCAAGGCAAACCTCTC-3′, Slug R, 5′-GTAT GACAGGCATGGAGTAACTCTC-3′. NRK-52E cells were transfected with the pcDNA3.1 or pcDNA-SRF plasmid and a nonspecific siRNA or the SRF-specific siRNA along with empty vector or pGL3-slug plasmids by using HiPerFect Transfection Reagent (Qiagen, Duesseldorf, Germany). Luciferase activities were evaluated through a luciferase assay system (Promega, Madison, Wisconsin, USA), and the luminescence was measured by using an illuminometer (BMG FLUOstar OPTIMA, Germany).

### Chromatin immunoprecipitation (ChIP) and semi-quantitative PCR

ChIP was performed as previously described [33]. Chromatin fragments were co-immunoprecipitated from UA-treated NRK-52E cells and control lysates with the appropriate amount of SRF antibody or an equivalent amount of rabbit IgG (8 mg) as a control. The purified DNA fragments were used as templates for PCR amplification. The DNA amplification procedure for semi-quantitative PCR was performed using 2×Taq PCR Master Mix (Tiangen, Beijing, China) according to the manufacturer’s protocol. PCR products were visualised on 1.5% agarose gels stained with GelRed (Biotium, Hayward, CA, USA) under UV transillumination. The specific primers were as follows: Slug (SRE1) F, 5′-CGTCTGTCTCCCTCACTGGAC-3′, Slug (SRE1) R, 5′-CTCTCGGCGGCTTGAAATGCC-3′, Slug (SRE2) F, 5′-GCCCGGGCTCTCACCGCCA-3′, and Slug (SRE2) R, 5′-GCAGCAGCGCCGCCAACTCCC-3′.

### Plasmids and transfection, quantitative RT-PCR, western blot and immunofluorescence staining

These methods were described previously [34]. All the primer sequences were reported in Table 2.

**Table 2:** The primers of quantitative RT-PCR.

**Gene**	**Forward (5′→3′)**	**Reverse (5′→3′)**
SRF	GCACAGACCTCACGCAGA	ATGTGGCCACCCACAGTT
ZO-1	GGAAACCCGAAACTGATGCTATGG	AACTGGCTGGCTGTACTGTGAG
Fibronectin	AGACCCCAGGCACCTATCAC	TCGGTCACTTCCACAAACTG
Slug	CATCTGCAGACCCACTCTGA	AGCAGCCAGACTCCTCATGT
GAPDH	GGATTTGGTCGTATTGGG	GATGATCTTGAGGCTGTTGTC

### Transwell chamber migration assay

The motility of NRK-52E cells was determined using a transwell chamber migration assay, as previously described [35].

### Statistical analysis

All data are shown as the mean ± SEM. Every experiment was repeated at least 3 times. Statistical analysis was performed by using SPSS (version 20.0; SPSS Inc., Chicago, IL, USA). Intergroup comparisons were carried out through one-way analysis of variance or an unpaired t-test. *P*<0.05 was considered statistically significant.

## Supplementary Material

Supplementary Materials
