# Supplementary material for: Crucial role of serum response factor in renal tubular epithelial cell epithelial-mesenchymal transition in hyperuricemic nephropathy
Source: Aging (Albany NY). 2019 Nov 27;11(22):10597–609. doi: 10.18632/aging.102479 (PMC6914413; doi:10.18632/aging.102479)
Supplement: Supplementary Materials [file aging-11-102479-s001..pdf]

SUPPLEMENTARY METHODS

Sample collection and biochemical analysis

At the end of the study, rats were weighed and housed in metabolic cages to collect 24-h urine samples. The rats were euthanized through an intraperitoneal injection of pentobarbital (50 mg/kg of BW), and then blood samples were collected from the left ventricle. Systemic perfusion with PBS through the left ventricle was carried out to wash out any remaining blood. The kidneys were quickly removed, decapsulated, weighed and dissected into two parts. One part was snap-frozen in liquid nitrogen and stored at −80°C for molecular biology studies, while the other part was stored in a 4% paraformaldehyde solution for histopathological analysis. Serum UA, serum creatinine (Scr), blood urea nitrogen (BUN) and serum albumin levels as well as 24-hour urinary albumin excretion (24-h UAE) were measured by the Department of Pathology at the Affiliated Hospital of Qingdao University.

Immunohistochemical staining

After deparaffinization, rehydration, and blockade of endogenous peroxidase activity, sections were microwaved in Tris-EDTA (pH 9.0) for 17 min for antigen retrieval, and then the sections were incubated overnight at 4 °C with a primary antibody. The sections were thoroughly rinsed with PBS, followed by incubation with a secondary antibody labeled with horseradish peroxidase (HRP) at room temperature for 40 min. The detection process was carried out by using diaminobenzidine and hematoxylin (ZSGB-BIO, Beijing, China). Omission of the primary antibody was used as a negative control to verify antibody specificity. Imaging of all sections was performed by using a Leica microscope electronic imager. All analyses were performed in a blinded fashion. Researchers were blind to which group samples were assigned to.

Plasmids and transfection

pcDNA3.1-SRF containing the full-length cDNA sequence of mouse SRF and control pcDNA3.1 vectors were gifted by Dr. Eric Olson (Department of Molecular Biology, UT Southwestern Medical Center at Dallas, USA). NRK-52E cells were transfected with these plasmids using Lipofectamine 2000 (Invitrogen, Carlsbad, CA, USA) according to the manufacturer's protocol. The infected cells were incubated for 5 h and then returned to complete medium. After 72 h, whole-cell lysates were collected for gene and protein expression analyses. The infected NRK-52E cells were submitted to immunofluorescence staining with an anti-SRF antibody as well.

Quantitative RT-PCR

Total RNA was isolated using TRIzol (Invitrogen, Carlsbad, CA, USA). First-strand cDNAs were synthesized from 2 µg of total RNA in a 20 µL reaction volume using a Prime Script RT reagent kit (TaKaRa Biotechnology, Dalian, China). Quantitative RT-PCR was performed with an Eppendorf Mastercycler ep realplex detection system (Eppendorf, Hamburg, Germany) with Faststart universal SYBR green master mix (Roche, Frankfurt, Germany) according to the manufacturer's protocol. The relative mRNA levels were normalized to the GAPDH level and calculated with the $2^{-\Delta\Delta CT}$ method. All the primer sequences are reported in Table 1.

Western blot

Kidney tissue samples or cell homogenates were lysed and stored at −80°C until assayed. Protein concentrations were determined using Coomassie reagent. Equal amounts of total protein (50 µg) were subjected to 12% sodium dodecyl sulfate-polyacrylamide gel electrophoresis (SDS-PAGE) and then transferred onto PVDF membranes (Millipore, Temecula, CA, USA). The blots were blocked for 1 h at room temperature with 5% skim milk in Tris-buffered saline (pH 7.4) containing 0.1% Tween 20 (TBST), followed by an incubation for 16 h at 4 °C with primary antibodies. After further washing with TBST, the blots were incubated with horseradish peroxidase (HRP)-conjugated secondary antibodies for 2 h at room temperature. Immune complexes were detected using enhanced chemiluminescence (Millipore, Temecula, CA, USA) and exposed to Kodak X-OMAT film. The immunoreactive bands were quantified by ImageJ analysis software (NIH, Bethesda, MD, USA).

Immunofluorescence staining

NRK-52E cells were fixed for 20 min at room temperature in 4% paraformaldehyde and then permeabilized with 0.3% Triton X-100 (Sigma) in PBS for 10 min. The fixed cells were incubated with a primary antibody against SRF, followed by the appropriate FITC-conjugated secondary antibody (ZSGB, Beijing, China). Nonimmune IgG served as a negative control, and no fluorescence was observed. To

visualize nuclei, the NRK-52E cells were incubated in a 1 μg/ml DAPI (Sigma, St. Louis, MO, USA) solution for 5 min. The slides were viewed under a Nikon epifluorescence microscope, and the expression of SRF was quantified by ImageJ analysis software.

Transwell chamber migration assay

The motility of NRK-52E cells was determined using a transwell chamber migration assay with tissue culture-treated polycarbonate membrane transwell filters (8-μm pore size; Corning, Cambridge, MA, USA) as previously described [23]. NRK-52E cells transfected with pcDNA3.1-SRF or empty pcDNA3.1 vectors were seeded into the upper chamber of the filters. After 48 h of incubation at 37 °C, the cells were fixed with methanol for 20 min and stained with 0.1% crystal violet for 20 min. The cells on the top side of the filters were carefully removed with a cotton swab. The cells that migrated to the underside of the filters were counted in 3 nonoverlapping 20× fields (at a magnification of 100×) and photographed under a Nikon microscope.
